# Super-Branched PdCu Alloy for Efficiently Converting Carbon Dioxide to Carbon Monoxide

**DOI:** 10.3390/nano13030603

**Published:** 2023-02-02

**Authors:** Kaili Bao, Yunjie Zhou, Jie Wu, Zenan Li, Xiong Yan, Hui Huang, Yang Liu, Zhenhui Kang

**Affiliations:** 1 Institute of Functional Nano & Soft Materials (FUNSOM), Jiangsu Key Laboratory of Advanced Negative Carbon Technologies, Soochow University, Suzhou 215123, China; 2 Macao Institute of Materials Science and Engineering (MIMSE), MUST-SUDA Joint Research Center for Advanced Functional Materials, Macau University of Science and Technology, Taipa, Macao 999078, China

**Keywords:** PdCu alloy, electrocatalytic CO_2_ reduction reaction, transient photovoltage, CO

## Abstract

The alloying of noble metals with Cu is one of the most effective strategies for improving catalytic performance and reducing cost in electrocatalytic carbon dioxide reduction reactions (CO_2_RR). Previous works usually focused on the influence of morphology and composition on the catalytic activity, but lacked the study of the valence state ratio of metals and the electron transfer behavior on alloys. In this work, PdCu−2 alloy (Pd/Cu molar ratio is 1:2) was obtained by a simple one-step solvothermal method, which can effectively convert CO_2_ to CO with a maximum Faradaic efficiency (FE) of 85% at −0.9 V (vs. RHE). Then, the effect of the chemical state of Pd and Cu on the catalytic performance was investigated. The X-ray photoelectron spectroscopy (XPS) shows that the binding energy of Pd in PdCu alloy has a negative shift, which has affected the adsorption of key intermediates. When the proportion of oxidized state and zero-valent metal in the alloy is about 1:2, the PdCu alloy shows the best catalytic activity. In addition, the transient photovoltage (TPV) measurements further demonstrate that due to the introduction of Cu, the electron transfer rate of PdCu−2 becomes the slowest, which helps the accumulation of electrons on PdCu−2 and leads to the improvement of catalytic performance for electrocatalytic CO_2_RR. This work can provide more insights into the alloy catalysts of electrocatalytic CO_2_RR.

## 1. Introduction

The excessive use of fossil fuels has produced a large amount of carbon dioxide (CO_2_), which has broken the original carbon balance of the earth’s ecological environment and led to the deterioration of the environment, such as global warming, extreme weather, land desertification, ocean acidification, etc. [1,2]. Therefore, it is very necessary to find efficient and green ways to consume and convert CO_2_. Compared to traditional thermal catalysis [3,4], electrocatalysis is a more efficient and green method of converting CO_2_ to valued chemicals [5,6,7,8]. Electrocatalytic carbon dioxide reduction reaction (CO_2_RR) has many advantages, such as mild reaction conditions, easy reactors, and high CO_2_ conversion efficiency [9,10,11]. However, the high overpotential of the reaction and the low activity and selectivity of the catalysts hinder the development of electrocatalytic CO_2_RR [12]. In addition, since the theoretical equilibrium reduction potential of various carbon products is very close to that of hydrogen evolution reaction (HER), HER will compete with CO_2_RR for electrons and protons, which will affect the Faradaic efficiency (FE) and selectivity of products [13]. Therefore, the design and synthesis of high-efficiency and low-cost catalysts have always been a research focus [14,15,16].

Because of its high activity and good conductivity, metal is the most commonly used electrocatalytic CO_2_RR catalyst. Gold (Au), sliver (Ag), palladium (Pd), and other noble metals have been widely studied due to their good selectivity of products [17,18,19,20]. However, the noble metals are expensive and not suitable for industrial production. Among numerous metal catalysts, copper (Cu) is the only metal that can produce more than a dozen hydrocarbon products and alcohols [21]. However, Cu-based catalysts often need to be regulated to meet the requirements of high activity, high selectivity, and low overpotential [22]. The catalytic performance of Cu-based catalysts can be effectively adjusted by adjusting the types, proportions, and chemical states of elements in Cu-based catalysts [23,24,25].

Alloying is a good strategy for adjusting the structure and chemical state of catalysts [26]. Due to the electronic effect and synergistic effect, it can effectively adjust the electronic structure, control the metal surface morphology, and adjust the binding strength of intermediate substances to accelerate the reaction rate on the catalyst surface [27,28]. In terms of electronic structure, alloying will affect the *d*-band center of metals, and then affect the adsorption energy of intermediates [29]. In addition, in terms of geometric effects, alloying may affect the surface strain and bonding mode of intermediates [30]. In previous works, alloying Pd and Cu can achieve higher catalytic activity and adjust the selectivity of products. Yin et al. [31] prepared carbon-supported PdCu alloy with a size less than 5 nm, and improved carbon monoxide (CO) selectivity by adjusting the size and composition of the catalyst. In addition, Chen et al. [32] regulated the morphologies and compositions of PdCu nanoalloys to obtain highly efficient electrocatalysts for the conversion of CO_2_ to CO. However, these works usually focused on the effect of size, morphology, and composition on catalytic performance. There is a lack of investigations about the valence state ratio of metals and the electron transfer behavior on alloys.

In this work, we synthesized the PdCu alloy catalysts with different Pd/Cu molar ratios to adjust the activity and selectivity of CO_2_RR. Alloying Pd and Cu effectively improves the FE on pure Pd, and changes the catalytic behavior of pure Cu. The CO_2_RR product of PdCu alloy is only CO, and PdCu−2 alloy has the best catalytic performance for converting CO_2_ to CO. The FE of CO can reach up to 85% at −0.9 V (vs. RHE) and the current density is about −7.76 mA cm^−2^. Compared with pure Pd, the FE of CO increased by 20%. The X-ray photoelectron spectroscopy (XPS) reveals that when the proportion of oxidized state and zero-valent metal in the alloy is about 1:2, the PdCu alloy (PdCu−2) shows the best electrocatalytic CO_2_RR performance. In addition, transient photovoltage (TPV) measurements indicate that the PdCu−2 alloy has the slowest electron transport rate and can accumulate more electrons on its surface for CO_2_ activation and CO_2_RR, which can effectively improve the activity and selectivity of catalysts.

## 2. Materials and Methods

### 2.1. Materials

Potassium chloropalladite (99.9%, K_2_PdCl_4_), Copper (II) acetate (Cu(OAc)_2_·H_2_O), ethylene glycol (98%, C_2_H_6_O_2_), and 1-methylimidazole, (≥99.0%, C_4_H_6_N_2_).

### 2.2. Synthesis of PdCu Alloy

The super-branched PdCu alloys were prepared by solvothermal process modified from prior reports [33]. 0.5 mmol K_2_PdCl_4_ and 1 mmol Cu(OAc)_2_·H_2_O were added into the mixed solution of 12 mL 1-methylimidazole and 50 mL ethylene glycol. The dispersed suspension was transferred into a 100 mL Teflon-lined stainless-steel autoclave after sonicating for 15 min. Then, the autoclave was maintained at 160 °C for 6 h in the oven. After the reaction system cooled down to room temperature naturally, the product was obtained by centrifuging and washed with water and ethanol three times. Finally, the black powder was dried at 60 °C in a vacuum oven for 12 h. According to the feed ratio of the reaction, this product was named as PdCu−2. 4 mmol and 0.25 mmol K_2_PdCl_4_ were used to synthesize the PdCu−1 and PdCu−3, respectively. The pure Pd and pure Cu were prepared as discussed in the above method without the addition of Cu(OAc)_2_·H_2_O and K_2_PdCl_4_, respectively.

### 2.3. Electrochemical Measurements

A total of 5 mg of catalyst was dispersed into 1 mL 0.5 wt.% Nafion water solution and ultra-sounded for 5 min to obtain uniform ink. The working electrode was carbon paper (1 cm × 1.5 cm) or glassy carbon electrode (diameter: 3 mm, GCE) covered with catalyst. An amount of 400 μL catalyst ink was dropped on the two sides of a carbon fiber paper, respectively, and dried at 60 °C to obtain the working electrode. A total of 6 µL catalyst dispersion solution was dropped on the glassy carbon electrode, and dried at room temperature.

Linear sweep voltammetry curves (LSV) were performed in the single cell from 0 V to −1.8 V (vs. SCE) with N_2_-saturated and CO_2_-saturated 0.5 M KHCO_3_ solution, respectively. The working electrode was glassy carbon electrode coated with catalysts, the reference electrode was the saturated calomel electrode (SCE), and the counter electrode was a platinum sheet. All potentials were referenced to a reversible hydrogen electrode (RHE) by adding a value of (0.241 + 0.059 × pH) V.

The electrocatalytic CO_2_RR performance measurements were carried out in a typical H-cell using three-electrode system. The electrolyte and gas in the two chambers of H-cell were separated by Nafion 212 proton exchange membrane. Each of the chambers was filled with 35.0 mL 0.5 M KHCO_3_ (saturated with CO_2_) and 30.0 mL of CO_2_ in the headspace. In order to activate the catalyst, 50 segments of cyclic voltammetry (CV) test from 0 V to −1.8 V (vs. SCE) were carried out before potentiostatic electrocatalysis. A constant voltage was applied to the working electrode for a period of time, and then the gas in the cathode chamber was extracted by a disposable syringe for further detection. Pana A60 gas chromatography was employed to detect and analyze gas-phase carbon products (CO, CH_4_, C_2_H_4_ and C_2_H_6_). The by-product H_2_ was detected by East–West Analysis gas chromatography. In addition, the liquid products were analyzed by 1H NMR (Bruker AVANCEAV III 400) spectrometer. An amount of 500 uL of electrolyte after reaction and 100 μL D_2_O and 0.05 μL dimethyl sulfoxide (DMSO, Sigma-Aldrich, St. Louis, American, 99.99%) were mixed as the internal standard. The FE of various products was calculated by the following formula:

(1) $$\mathrm{FE}=\frac{z\mathrm{nF}}{Q}\;\times \;100\%,$$

where *z* is the number of electrons transferred, F is the Faraday constant (96,485 C mol^−1^), n is the number of moles of the produced products, and *Q* is the total charge passed.

## 3. Results and Discussion

### 3.1. Characterizations of Catalyst

**Figure 1:**
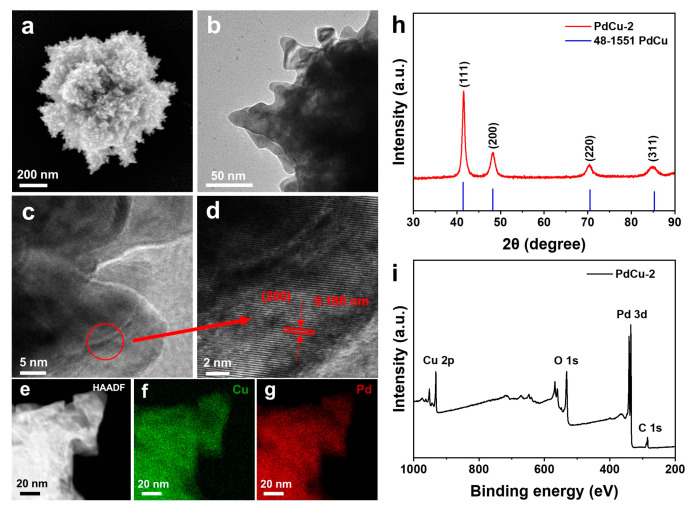
Characterization of PdCu−2: (**a**) SEM image of PdCu−2; (**b**) TEM image of PdCu−2; (**c**,**d**) HRTEM images of PdCu−2; (**e**–**g**) Elemental mapping images of Pd and Cu of PdCu−2; (**h**) XRD pattern of PdCu−2; (**i**) The full survey XPS spectrum of PdCu−2.

The PdCu alloy was synthesized by a one-pot solvothermal method. Inductively Coupled Plasma Optical Emission Spectrometer (ICP-OES) was used to study the metal ratio in the PdCu alloy (Table S1). The mass ratio of Pd:Cu in PdCu−1 is 83:17, and the mass ratio of Pd:Cu in PdCu−2 is 60:40. The PdCu−3 is 49:51. As shown in Figure 1a, the PdCu−2 shows a 3D multi-branched sea cucumber structure. The average size of PdCu−2 is about 500 nm. The length and width of branches are between 50 and 100 nm and 20 and 50 nm, respectively (Figure 1b). High-resolution transmission electron microscopy (HRTEM) was used to further characterize the detailed structural characteristics of the branches. Clear lattice stripes can be observed in Figure 1d. The crystal plane spacing is 0.188 nm, which corresponds to the (200) plane of PdCu alloy. This proves that the prepared catalyst is PdCu alloy. Figure 1e–g shows the EDS mapping image of PdCu−2. According to the element distribution of EDS mapping, Pd and Cu are evenly distributed, which confirms that 3D sea cucumber-like nanoparticles are composed of uniform PdCu alloy.

The morphology of Pd, PdCu−1, PdCu−3, and Cu are presented in Figures S1 and S2 (Supplementary Materials). These images indicate that the amount of added Cu(OAc)_2_·H_2_O has a great influence on the morphology of the catalysts. The scanning electron microscopy (SEM) and transmission electron microscopy (TEM) images of pure Pd show a 3D structure of slender branches stretching from the central axis to the periphery (Figures S1a,b and S2a,b). With the incorporation of Cu (the molar ratio of Pd:Cu is 4:1), the branches become thicker, shorter, and denser. When the amount of Cu(OAc)_2_·H_2_O continues to increase (the molar ratio of Pd:Cu is 1:4), the morphology of PdCu−3 in Figures S1c,d and S2c,d is very similar to PdCu−2, but its branches become shorter and denser. These SEM and TEM images show that the introduction of Cu has a great influence on the morphology of the catalyst. The morphology of pure Cu is shown in Figures S1g,h and S2g,h. The pictures indicate that pure Cu has an uneven polyhedral particle structure, and there are no branches on the surface of pure Cu. The above results illustrate that in this experimental system, Pd is the main body in forming a 3D branched structure, and the incorporation of Cu can effectively affect the growth of the catalyst and change the final morphology.

X-ray powder diffraction (XRD) was used to analyze the phase and crystal structure of the catalyst. Figure 1h shows the XRD diagram of the PdCu−2 alloy, and the XRD diagrams of pure Pd, PdCu−1, PdCu−3, and pure Cu are shown in Figure S3. There are four obvious characteristic peaks at 2θ = 41.42°, 48.21°, 70.54°, and 85.29°, which correspond to the (111), (200), (220), and (311) crystal planes of PdCu (JCPDS No. 48-1551). There are no other diffraction peaks in PdCu−2, which indicates that the single-phase PdCu alloy is formed. As shown in Figure S3, the peaks of pure Pd and Cu correspond to the standard peaks of Pd and Cu (JCPDS No. 05-0681 and 01-1241), respectively. Furthermore, with the increase in Cu content, the characteristic diffraction peak of PdCu alloy gradually moves to a higher angle, indicating that the incorporation of Cu makes the lattice shrink and PdCu alloy was formed. The full survey XPS spectrum shows that the main elements in the PdCu−2 alloy are Pd and Cu.

**Figure 2:**
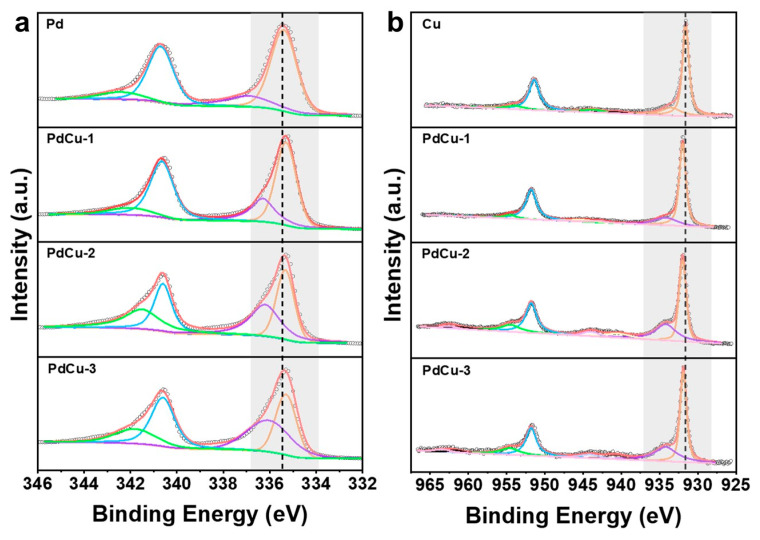
XPS spectrum of Pd, PdCu−1, PdCu−2, PdCu−3, and Cu: (**a**) high-resolution XPS spectrum of Pd 3d for Pd, PdCu−1, PdCu−2, and PdCu−3; (**b**) high-resolution XPS spectrum of Cu 2p for Cu, PdCu−1, PdCu−2, and PdCu−3.

In order to further investigate the effect of alloying on the surface chemical state of catalysts, the high-resolution XPS spectra were performed. Figure 2a is the high-resolution XPS spectra of Pd 3d for pure Pd, PdCu−1, PdCu−2, and PdCu−3. The high-resolution XPS spectra of Cu 2p for pure Cu, PdCu−1, PdCu−2, and PdCu−3 are displayed in Figure 2b. The peaks at 335.4 eV and 340.7 eV of pure Pd correspond to the Pd^0^ of the Pd 3d_5/2_ and 3d_3/2_ orbitals, while the Pd^2+^ of Pd 3d_5/2_ and 3d_3/2_ orbitals are centered at 336.9 eV and 342.4 eV. With the incorporation of Cu, the binding energy of Pd^0^ of the Pd 3d_5/2_ and 3d_3/2_ orbitals shows a negative shift (≈−0.1 eV). The peaks of Pd^2+^ also present similar shifts for all the alloys. For PdCu−2, the Pd^0^ and Pd^2+^ of the Pd 3d_5/2_ and 3d_3/2_ orbitals are located at 335.3 eV, 336.2 eV, 340.6 eV, and 341.5 eV, respectively. Accordingly, the peak position of Cu also has a corresponding positive shift for all the alloys. As shown in Figure 2b, the Cu 2p spectrum can be divided into two peaks. The binding energy of the Cu 2p at 931.6 eV is assigned to Cu^0^ of the Cu 2p_1/2_ orbital, and the Cu^2+^ peak of Cu 2p_1/2_ is centered at 933.5 eV. Compared with pure Cu, the position of Cu^0^ of the Cu 2p_1/2_ orbital in PdCu−2 shows a positive shift of +0.4 eV, which is located at 932.0 eV. In the same way, the position of Cu^2+^ of the Cu 2p_1/2_ orbital in PdCu−2 is 934.2 eV, which experienced a positive shift of +0.7 eV. The reduction of binding energy can be attributed to the strain and ligand effect. The tensile strain increases the distance between Pd atoms, reduces the overlap of d orbitals, and affects the bandwidth. In addition, as shown in Tables S2 and S3, with the increase in Cu content, the content of oxidized state Pd and Cu also increases. The ratio of oxidized state Pd and zero-valent Pd is nearly 1:2, and the ratio of that in Cu is also nearly 1:2. The increase in oxidized state Pd indicates the downshift of d-band center, which can enhance the adsorption energy of *COOH and promote the desorption of CO [26,32,34]. High-resolution XPS spectra show that there is a certain electronic interaction between Pd and Cu. Because Pd is more electronegative, electrons tend to transfer from Cu to Pd [33,35]. Alloying makes the electronic structure change obviously, which has a crucial effect on the adsorption energy of intermediates and the catalytic performance of catalysts [36,37].

### 3.2. Electrocatalytic Activity of Catalysts

**Figure 3:**
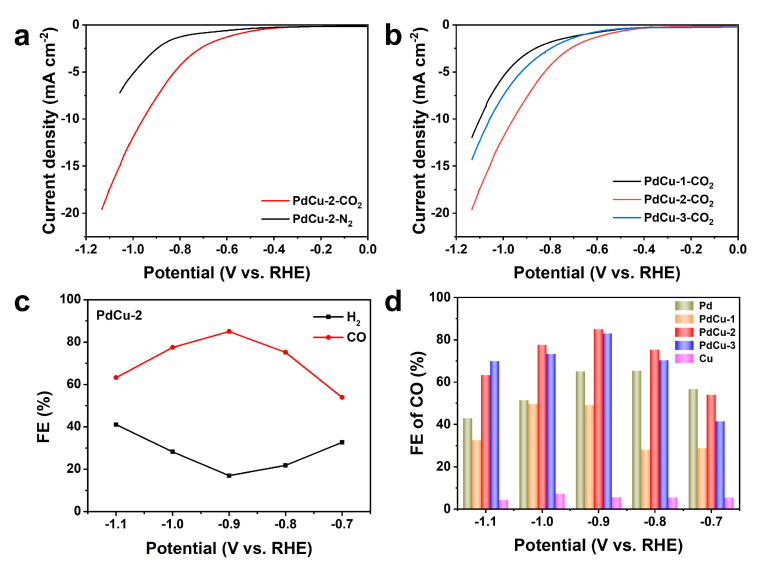
LSV curves and FE of the catalysts: (**a**) LSV curves of PdCu−2 in CO_2_ (red line) and N_2_-saturated (black line) 0.5 M KHCO_3_ solution; (**b**) LSV curves of PdCu−1 (black line), PdCu−2 (red line), and PdCu−3 (blue line) in CO_2_-saturated 0.5 M KHCO_3_ solution; (**c**) FE of CO and H_2_ on PdCu−2 at the applied potentials; (**d**) FE of CO on Pd, PdCu−1, PdCu−2, PdCu−3, and Cu at the applied potentials.

In order to evaluate the electrocatalytic CO_2_RR performance of PdCu alloys, linear sweeping voltammetry (LSV) tests were performed. As shown in Figure 3a, the current density of PdCu−2 in CO_2_-saturated 0.5 M KHCO_3_ solution is much larger than that in N_2_-saturated 0.5 M KHCO_3_ solution, which is mainly due to the occurrence of CO_2_RR. The initial potential of PdCu−2 in CO_2_-saturated electrolyte is −0.56 V (vs. RHE), while the initial potential in N_2_-saturated electrolyte increases to −0.75 V. In addition, the current density obviously increases from −2.55 mA cm^−2^ to −7.76 mA cm^−2^ at −0.9 V (vs. RHE). Then, the comparison of the LSV curves of PdCu−1, PdCu−2, and PdCu−3 in the CO_2_ atmosphere shows that the current density of PdCu−2 is the largest and the initial potential of that is the smallest (Figure 3b), which means that the CO_2_RR performance of PdCu−2 is the best. The LSV curves of PdCu−1 and PdCu−2 in the N_2_ and CO_2_ atmosphere are exhibited in Figure S4. Both of them show higher current densities in CO_2_-saturated electrolytes. Most of the increase in the current density comes from the occurrence of CO_2_RR, which can be proved by further product analysis.

The electrolysis experiments were conducted in a typical H-type cell. The gas products were analyzed by gas chromatography, and the liquid products were analyzed by ^1^H NMR. Only gas products were observed in this reaction system. The FEs of CO and H_2_ on PdCu−2 are shown in Figure 3c, and the FEs of carbon products and H_2_ on Pd, PdCu−1, PdCu−3, and Cu are displayed in Figure S5. From these results, there are only gas products (CO and H_2_) in the reaction system of Pd and PdCu alloys. PdCu−2 shows the best activity and selectivity of CO_2_RR among all the catalysts, which is consistent with the results of LSV curves. The FE of CO on PdCu−2 increases from 53.9% at −0.7 V (vs. RHE) to 85% at −0.9 V, and then decreases with the increase in applied potential. As Figure 3d shows, the pure Pd has the maximum FE_CO_ of 65.31% at −0.8 V, while the FE of CO on PdCu−1 decreases gradually due to the incorporation of a small amount of Cu. Then, when the contents of Pd and Cu reach a balance (PdCu−2), the FE of CO reaches the maximum, which is better than some reported CO_2_RR electrocatalysts (as Table S4 shows in the Supplementary Materials). However, with the increase in Cu content, excessive Cu will affect the adsorption energy of *CO [38], and the FE of CO will decrease accordingly. For pure Cu, there are three kinds of carbon products in the reaction system, and the FE and selectivity are very poor. Therefore, the alloying can effectively improve the catalytic activity and selectivity.

**Figure 4:**
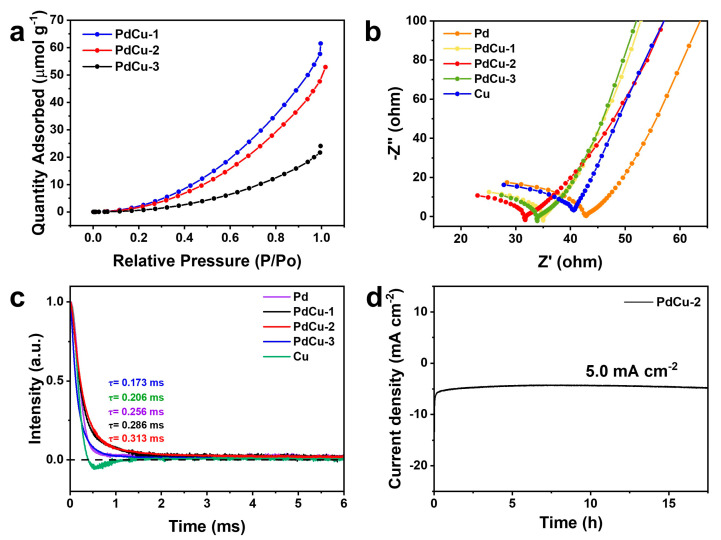
(**a**) The CO_2_ adsorption isotherms of PdCu−1, PdCu−2, and PdCu−3; (**b**) EIS spectra of Pd, PdCu−1, PdCu−2, PdCu−3, and Cu; (**c**) TPV curves of Pd, PdCu−1, PdCu−2, PdCu−3, and Cu; (**d**) the stability test of PdCu−2 at the potential of −0.9 V (vs. RHE) for 17 h.

In order to explore the mechanism of CO_2_RR on PdCu alloy, a CO_2_ adsorption experiment was conducted. As shown in Figure 4a, PdCu−1 has the largest CO_2_ adsorption capacity, followed by PdCu−2 and PdCu−3. This may be caused by the gradual decrease in Pd content. Then, the electrochemical active surface area of the catalysts was analyzed by measuring the double-layer capacitance (*C_dl_*). Based on the Randles–Sevcik equation [39], the relationship between capacitance current density and scanning speed is calculated, and the results are shown in Figure S6d. It can be seen from the measured *C_dl_* values that PdCu−2 has the largest electrochemical active surface area, which is conducive to improving the electrocatalytic CO_2_ reduction rate and activity. In addition, the BET surface area of PdCu−2 is the biggest among all the catalysts (13.9592 m^2^ g^−1^). At the same time, the capacity of charge transfer rate at the electrode/electrolyte interface was analyzed by electrochemical impedance spectroscopy. Nyquist plots were obtained by testing under open circuit potential (Figure 4b), and the equivalent circuit (R (C(RW))) was used to fit the high-frequency and intermediate frequency band data of electrochemical impedance data. The results show that the charge transfer resistances (Rct) of PdCu alloys are much lower than that of pure metals (Pd and Cu), which can be attributed to the smaller particle size of the catalyst and the synergistic effect between metals. PdCu−2 has the smallest semi-circle radius (32 Ω) among all the PdCu alloys. Therefore, alloying can effectively adjust the electronic structure, facilitate the transfer of electrons from the electrode to the catalyst surface, and further promote the activation of CO_2_ to form intermediates, which is crucial for electrocatalytic CO_2_RR [40,41].

In order to further study the charge transfer on catalysts, the TPV test was performed. Figure 4c is the typical TPV attenuation curves of Pd, PdCu−1, PdCu−2, PdCu−3, and Cu. It can be seen that the attenuation trend of catalysts other than Cu is similar, decreasing from the highest point to zero with the increasing time. The TPV curve of PdCu−3 drops to the baseline fastest, while PdCu−2 is the slowest. Additionally, this may be helpful to the formation of multi-carbon products on the Cu surface. Then, the time decay constant (τ) values were calculated to compare the electron release and transfer velocities. The higher the τ is, the longer the charge retention time is, and the slower the charge transfer is [42]. The τ values of PdCu−3, Cu, Pd, PdCu−1, and PdCu−2 are 0.173 ms, 0.203 ms, 0.256 ms, 0.286 ms, and 0.313 ms, respectively. Among all the catalysts, the τ value of PdCu−2 is the biggest, which indicates that the electrons can stay longer on the surface of the PdCu−2 alloy. Higher electron concentration around the local environment means that more electrons can participate in the process of CO_2_ activation and subsequent CO_2_ reduction to CO.

At last, the stability experiment was carried out to prove good service life. As Figure 4d shows, the current density of the PdCu−2 catalyst can maintain around—−5 mA cm^−2^ for more than 17 h at the electrolysis potential of −0.9 V. Then, SEM images and the XRD pattern of PdCu−2 after the reaction are displayed in Figures S7 and S8. The morphology of PdCu−2 has no significant change, except that the surface has become slightly rough. In addition, the four characteristic diffraction peaks of PdCu−2 are slightly weaker.

**Figure 5:**
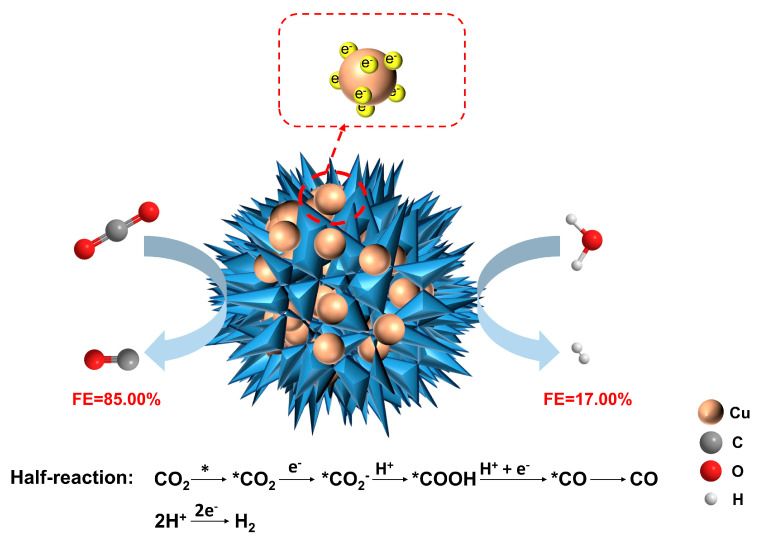
The mechanism of converting CO_2_ to CO on PdCu−2. (Here, the * is used as an indicator of activated substrates or surfaced attached species in the work).

A schematic diagram of the mechanism of converting CO_2_ to CO on the PdCu−2 alloy is presented in Figure 5. According to the SEM images of PdCu alloys with different ratios, Cu grows on the 3D branched structure with Pd as the main body. Firstly, because Pd has a stronger adsorption capacity for CO_2_, CO_2_ tends to be adsorbed on Pd. Then, because Cu has a good ability to store electrons, most of the electrons will be concentrated on the surface of Cu. These electrons were used to activate CO_2_ to *CO_2_. After that, *CO_2_ continues to catch electrons and protons to form *COOH. The next intermediate is *CO, which also needs one electron and one proton to generate. Finally, the *CO was desorbed from the surface of the PdCu−2 alloy. The smaller resistance of the PdCu−2 alloy helps the electrons transfer to the surface of the electrode quickly, which is beneficial to the activation of CO_2_. The proper ratio of oxidized state Pd and zero-valent Pd in the PdCu−2 alloy also adjusts the adsorption capacity of the catalyst for intermediates. In addition, alloying adjusted the electronic structure of pure metals, and the PdCu−2 alloy has the slowest electron relaxation rate. There are more electrons accumulated on the surface of the PdCu−2 alloy for CO_2_ activation and reduction. The energy barrier of *CO protonation is increased by the incorporation of Cu, which promotes the selectivity of products.

## 4. Conclusions

In this work, the PdCu alloy with the best CO_2_RR performance was obtained by adjusting the feeding ratio. The PdCu−2 alloy (feed molar ratio is 1:2) displays the best catalytic activity and selectivity for the conversion of CO_2_ to CO. The maximum FE of CO is 85% at −0.9 V (vs. RHE), and the current density can reach −7.76 mA cm^−2^. XPS spectra indicate that the alloying leads to the negative shift of binding energy of Pd and the increase in the ratio of oxidized state Pd and zero-valent Pd. It can enhance the adsorption of *COOH and promote the desorption of *CO. In addition, the TPV results demonstrate that the slowest electron transfer rate helps the accumulation of electrons on PdCu−2, which leads to the enhancement of catalytic performance for electrocatalytic CO_2_RR. The strategy of PdCu alloys can effectively reduce the cost of noble metal catalysts, which is very beneficial in terms of economic feasibility. In addition, the work can provide more insights into the investigations of the electron transfer behavior on catalysts.

## Data Availability

All the data generated or analyzed in this manuscript are available in the article.

## Supplementary Materials

The following supporting information can be downloaded at https://www.mdpi.com/article/10.3390/nano13030603/s1. Table S1: The molar ratio of Pd and Cu in PdCu alloys determined by the feed ratio of precursors and the mass ratio of Pd and Cu in PdCu alloys determined by ICP-OES. Table S2: The content ratio of the Pd^2+^ and Pd^0^ in pure Pd and PdCu alloys. Table S3: The content ratio of the Cu^2+^ and Cu^0^ in pure Cu and PdCu alloys. Table S4: Comparison of different catalysts in electrochemical CO_2_RR for CO formation. Figure S1: SEM images of Pd, PdCu−1, PdCu−3, and Cu. (a), (b) The SEM images of Pd. (c), (d) The SEM images of PdCu−1. (e), (f) The SEM images of PdCu−3. (g), (h) The SEM images of Cu. Figure S2: TEM images of Pd, PdCu−1, PdCu−3, and Cu. (a), (b) The TEM images of Pd. (c), (d) The TEM images of PdCu−1. (e), (f) The TEM images of PdCu−3. (g), (h) The TEM images of Cu. Figure S3: The XRD pattern of Pd, Cu, PdCu−1, PdCu−2, and PdCu−3. Figure S4: (a) LSV curves of PdCu alloys in CO_2_ and N_2_-saturated 0.5 M KHCO_3_ solution; (b) LSV curves of PdCu−1 in CO_2_ (red line) and N_2_-saturated (black line) 0.5 M KHCO_3_ solution; (c) LSV curves of PdCu−3 in CO_2_ (red line) and N_2_-saturated (black line) 0.5 M KHCO_3_ solution. Figure S5: FEs. (a) FE of CO and H_2_ on Pd; (b) FE of CO and H_2_ on PdCu−1; (c) FE of CO and H_2_ on PdCu−3. (d) FE of carbon products and H_2_ on Cu. Figure S6: Cyclic voltammetry obtained at various scan rates of (a) PdCu−1; (b) PdCu−2; (c) PdCu−3; (d) *C_dl_* of PdCu−1, PdCu−2, and PdCu−3 in 0.5 M KHCO_3_ aqueous electrolyte. Figure S7: (a) The SEM image of PdCu−2 before reaction; (b) the SEM image of PdCu−2 after reaction. Figure S8: The XRD pattern of PdCu−2 after reaction. References [43,44,45,46,47,48,49] are cited in the supplementary materials.
