# Supplementary material for: Super-Branched PdCu Alloy for Efficiently Converting Carbon Dioxide to Carbon Monoxide
Source: Nanomaterials (Basel). 2023 Feb 2;13(3):603. doi: 10.3390/nano13030603 (PMC9921487; doi:10.3390/nano13030603)
Supplement: Supplementary file 1 [file nanomaterials-13-00603-s001.zip › nanomaterials-2153650-supplementary.pdf]

Supplementary materials for

Super-branched PdCu alloy for efficiently converting carbon dioxide to carbon monoxide

Kaili Bao [1], Yunjie Zhou [1], Jie Wu [1], Zenan Li [1], Xiong Yan [1], Hui Huang [1 *], Yang Liu [1 *] and Zhenhui Kang [1, 2]

[1] Institute of Functional Nano & Soft Materials (FUNSOM), Jiangsu Key Laboratory of Advanced Negative Carbon Technologies, Soochow University, Suzhou, 215123, Jiangsu, PR China

[2] Macao Institute of Materials Science and Engineering (MIMSE), MUST-SUDA Joint Research Center for Advanced Functional Materials, Macau University of Science and Technology, Taipa 999078, Macao, China

* Correspondence: hhuang0618@suda.edu.cn; yangl@suda.edu.cn

1. Experimental Section

1.1. Instruments

The crystal structure of the catalysts was characterized by X-ray diffraction (XRD) using an X'Pert-ProMPD (Holand) D/max-γAX-ray diffractometer with Cu Kα radiation (λ = 0.154178 nm). Scanning electron microscopy (SEM) images were obtained with a ZEISS G500 scanning electron microscope with an acceleration voltage of 20 kV. Transmission electron microscope (TEM) and high-resolution TEM (HRTEM) images were obtained with a FEI-Tecnai F20 (200kV) instrument. X-ray photoelectron spectroscopy (XPS) was obtained by using a KRATOS Axis ultra-DLD X-ray photoelectron spectrometer with a monochromated Mg Kα X-ray source (hν = 1283.3 eV). The CO_2 adsorption was determined by plotting the adsorption isotherm of CO_2 at 25°C obtained using a Micromeritics ASAP 2050 instrument. The electrocatalysis actions were tested by a Model CHI 760C workstation (CH Instruments, Chenhua, Shanghai, China).

1.2. Transient photovoltage (TPV) measurements

The TPV measurements were conducted under room temperature on platinum net covered with power sample (1 cm×1 cm) as the working electrodes and Pt wire as the counter electrodes. The TPV was excited with a nanosecond laser radiation pulse (wavelength of 355 nm and the repetition rate was 5 Hz) from a third harmonic Nd : YAG laser (Beamtech Optronics Co., Ltd.). The TPV signals were amplified by an amplifier and were recorded by an oscilloscope. All measurements were performed at room temperature and under ambient pressure.

2. Supplementary Tables and Figures

Table S1. The molar ratio of Pd and Cu in PdCu alloys determined by the feed ratio of precursors and the mass ratio of Pd and Cu in PdCu alloys determined by ICP-OES.

Catalyst	Molar ratio (Pd : Cu)	Mass ratio (Pd : Cu)
PdCu-1	4 : 1	83 : 17
PdCu-2	1 : 2	60 : 40
PdCu-3	1 : 4	49 : 51

Table S2. The content ratio of the Pd^{2+} and Pd^0 in pure Pd and PdCu alloys.

Ratio	Pd^{2+}/Pd^0
Pd	1 : 3.6
PdCu-1	1 : 2.5
PdCu-2	1 : 1.8
PdCu-3	1 : 0.9

Table S3. The content ratio of the Cu^{2+} and Cu^0 in pure Cu and PdCu alloys.

Ratio	Cu^{2+}/Cu^{0}
Cu	1 : 5.6
PdCu-1	1 : 3.5
PdCu-2	1 : 1.9
PdCu-3	1 : 1.5

Table S4. Comparison of different catalysts in electrochemical CO_2RR for CO formation.

Catalysts	Products	Faradaic efficiency	Cell	Ref.
PdCu alloy	CO	85%	H-cell	This work
Ordered CuPd bimetal	CO	80%	H-cell	[43]
PdCu alloy	CO	82%	GDE-cell	[44]
M-Pd_7Cu_3	CO	80%	H-cell	[45]
FL-Pd_3Cu	CO	82.1%	H-cell	[46]
CuIn	CO	68.25	H-cell	[47]
Cu_9Au_{91}	CO	75%	H-cell	[48]
PdTe/CeO_2	CO	84%	H-cell	[49]

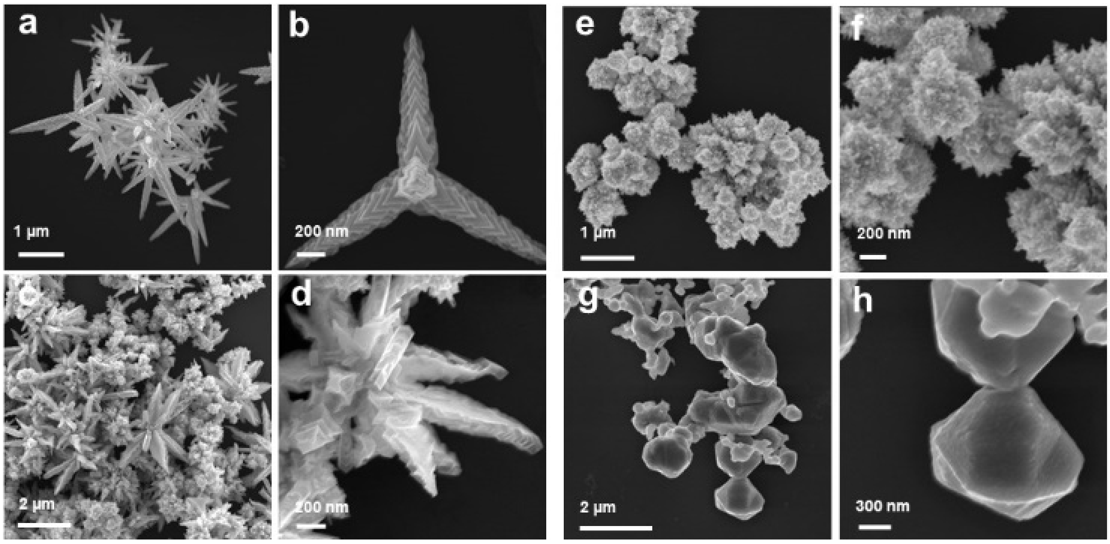

Figure S1. SEM images of Pd, PdCu-1, PdCu-3, and Cu. (**a**), (**b**) The SEM images of Pd. (**c**), (**d**) The SEM images of PdCu-1. (**e**), (**f**) The SEM images of PdCu-3. (**g**), (**h**) The SEM images of Cu.

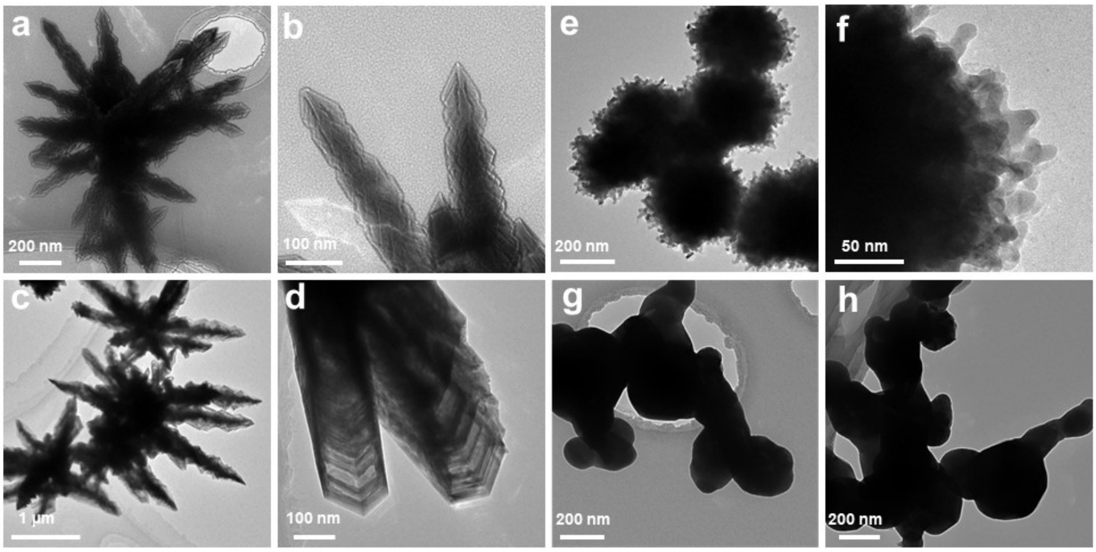

Figure S2. TEM images of Pd, PdCu-1, PdCu-3, and Cu. (**a**), (**b**) The TEM images of Pd. (**c**), (**d**) The TEM images of PdCu-1. (**e**), (**f**) The TEM images of PdCu-3. (**g**), (**h**) The TEM images of Cu.

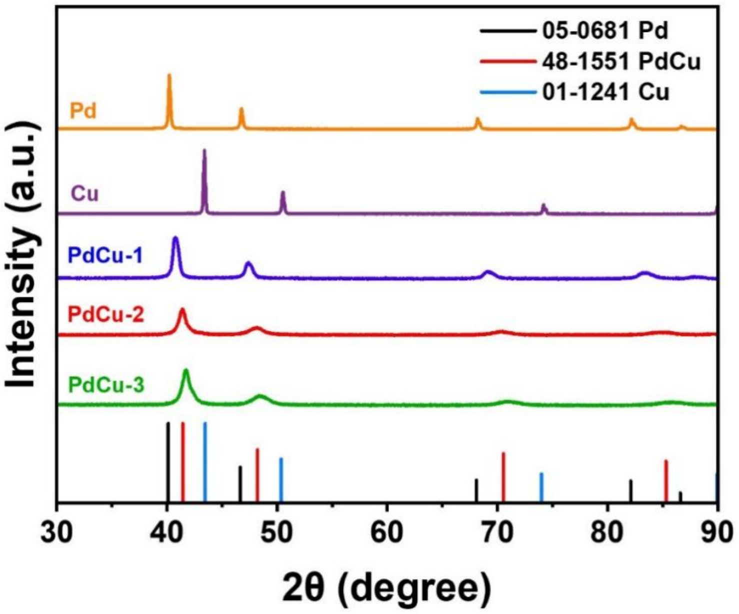

Figure S3. The XRD pattern of Pd, Cu, PdCu-1, PdCu-2, and PdCu-3.

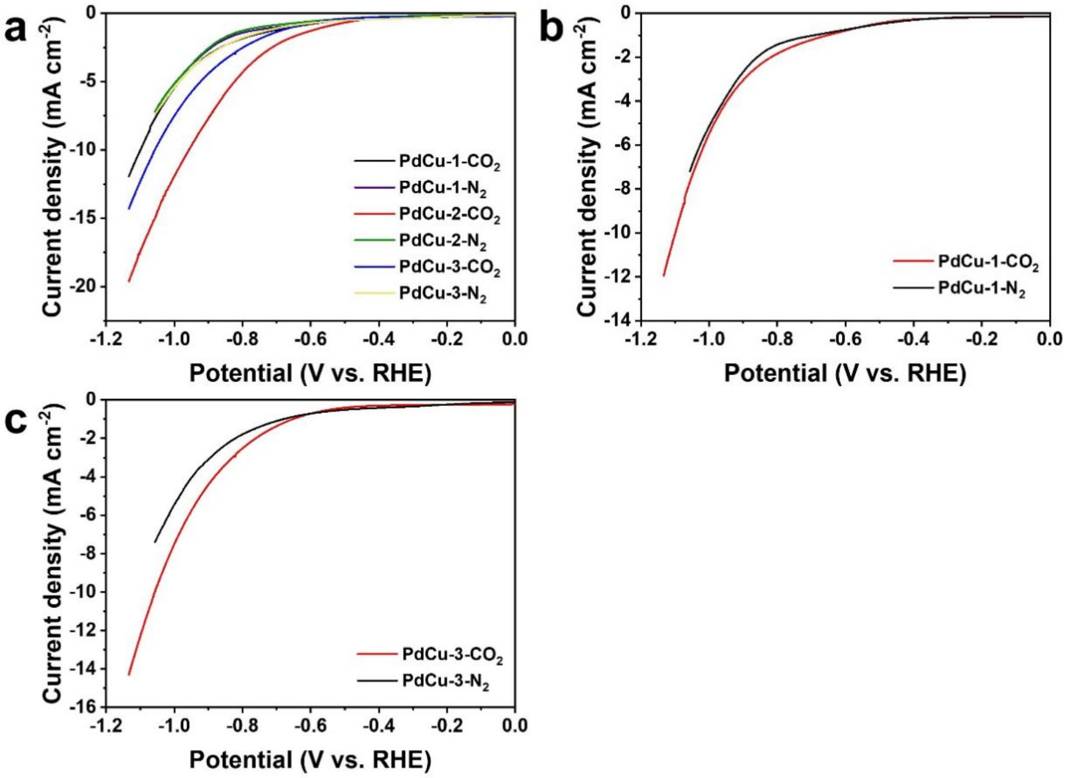

Figure S4. (**a**) LSV curves of PdCu alloys in CO_2 and N_2-saturated 0.5 M $KHCO_3$ solution; (**b**) LSV curves of PdCu-1 in CO_2 (red line) and N_2-saturated (black line) 0.5 M $KHCO_3$ solution; (**c**) LSV curves of PdCu-3 in CO_2 (red line) and N_2-saturated (black line) 0.5 M $KHCO_3$ solution.

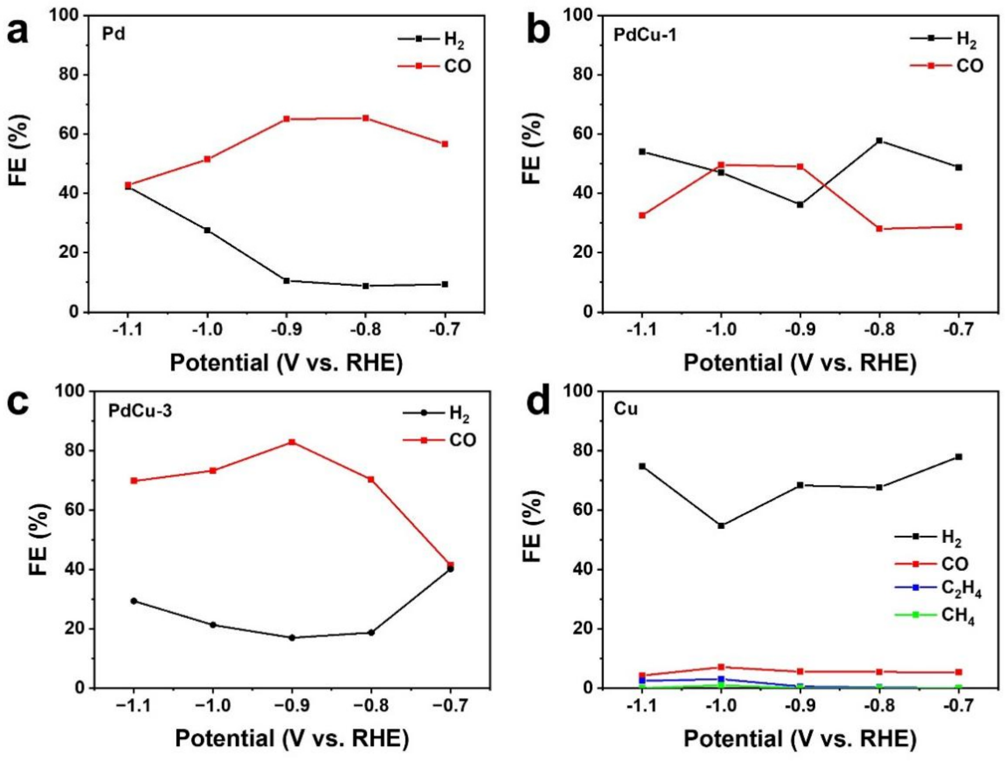

Figure S5. FEs. (**a**) FE of CO and H_2 on Pd; (**b**) FE of CO and H_2 on PdCu-1; (**c**) FE of CO and H_2 on PdCu-3. (**d**) FE of carbon products and H_2 on Cu.

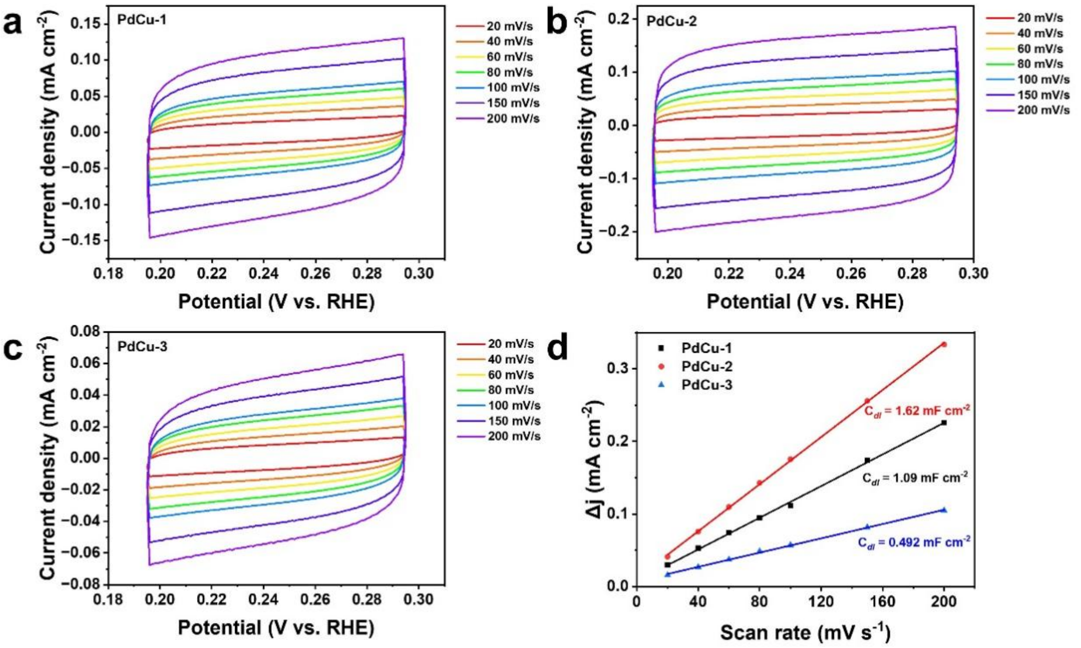

Figure S6. Cyclic voltammetry obtained at various scan rates of (**a**) PdCu-1; (**b**) PdCu-2; (**c**) PdCu-3; (**d**) C_{dl} of PdCu-1, PdCu-2, and PdCu-3 in 0.5 M $KHCO_3$ aqueous electrolyte.

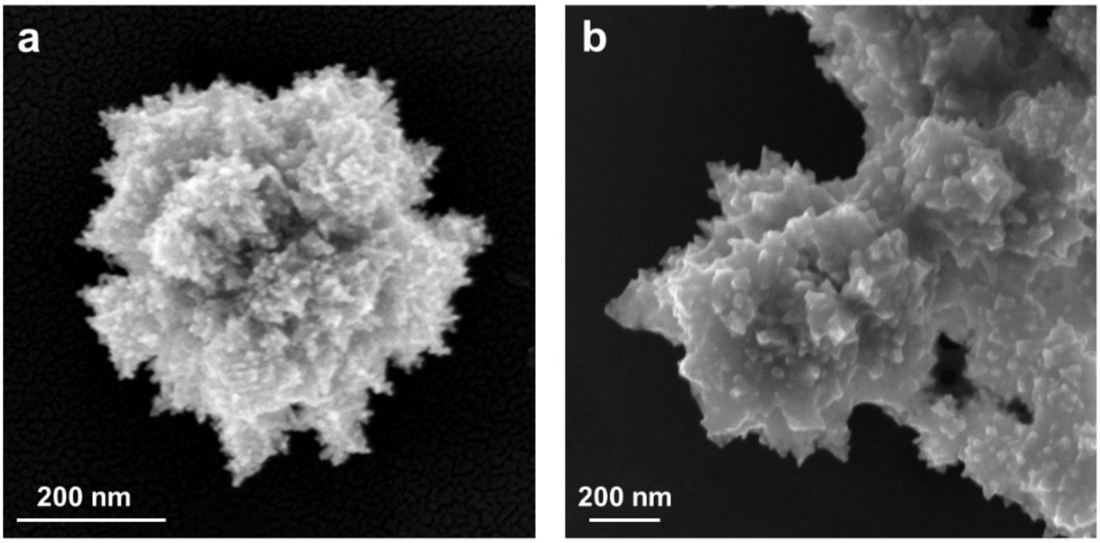

Figure S7. (**a**) The SEM image of PdCu-2 before reaction; (**b**) the SEM image of PdCu-2 after reaction.

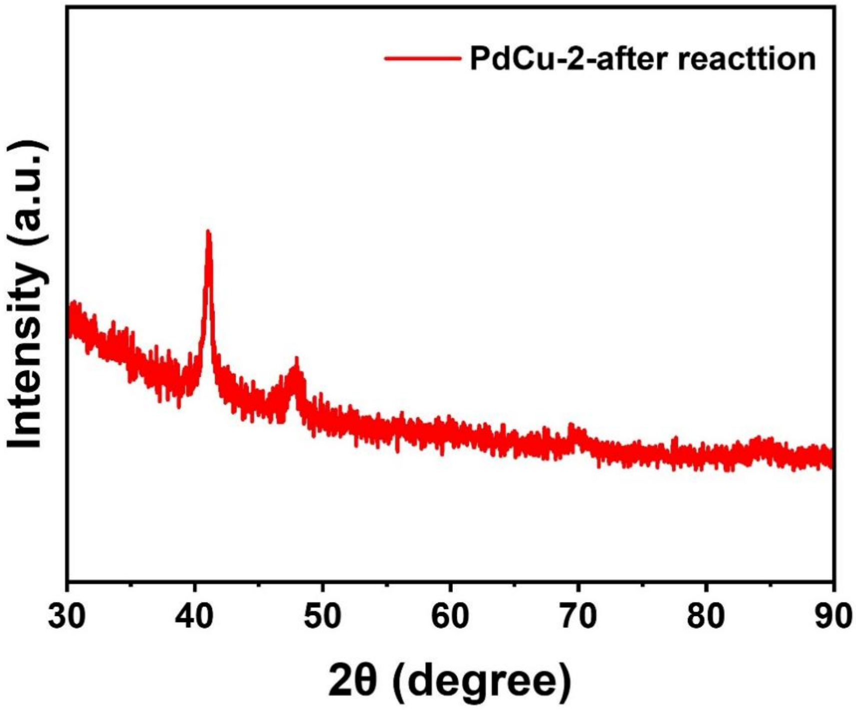

Figure S8. The XRD pattern of PdCu-2 after reaction.
